# Use of Deproteinized Bovine Bone in Association with Calcium Sulphate for Alveolar Socket Preservation

**DOI:** 10.3390/jcm14010003

**Published:** 2024-12-24

**Authors:** Annarita Signoriello, Alessandro Zangani, Paolo Faccioni, Elena Messina, Alessia Pardo, Giovanni Corrocher, Massimo Albanese, Giorgio Lombardo

**Affiliations:** Dentistry and Maxillo-Facial Surgery Unit, Department of Surgery, Dentistry, Paediatrics and Gynaecology (DIPSCOMI), University of Verona, Piazzale L.A. Scuro 10, 37134 Verona, Italy; alessandro.zangani@univr.it (A.Z.); paolo.faccioni@univr.it (P.F.); elena.messina@univr.it (E.M.); giovanni.corrocher@univr.it (G.C.); massimo.albanese@univr.it (M.A.); giorgio.lombardo@univr.it (G.L.)

**Keywords:** biomaterial, calcium sulphate, deproteinized bovine bone, extraction, regenerative, socket preservation

## Abstract

**Background:** The aim of this retrospective study was to compare the histomorphometry of post-extractive sites previously grafted with deproteinized bovine bone, with or without the association of a calcium sulphate preparation. **Methods:** The retrospective evaluation comprehended patients previously selected and treated for the extraction of one or more mono-radicular teeth, followed by an implant-prosthetic rehabilitation. Post-extractive sites had been randomly assigned to test or control group, respectively, if deproteinized bovine bone was used in association with a calcium sulphate preparation or alone. In both cases, a collagen membrane was employed to cover the grafted area. After four months, a biopsy of regenerated bone was taken from all grafted sites and then processed for histomorphometric analysis. **Results:** Of 24 samples analyzed 4 months after extraction, vital bone was present in 62.5% of cases for the test group and in 31.25% for the control group. Acellular bone was respectively found in 5% of cases for the test group and in 32.91% for the control group. Both these differences were statistically significant (*p* < 0.05) between groups. **Conclusions:** Calcium sulphate in association with deproteinized bovine bone seems to promote proper vital bone formation, with less acellular bone compared to deproteinized bovine bone used alone. Socket preservation procedures with the use of specific osteoconductive materials improve the maintenance of width and height of remaining bone. Findings of the present study offer clinicians a predictable protocol for preserving vital bone in early healing of post-extraction sites, slowing down the resorption process at the same time.

## 1. Introduction

Physiological changes regarding alveolar bone resorption after dental extraction are widely documented in literature [1,2,3]. Relevant variations are generally observed during the first year after extraction and more intensely during the first three months [4,5]. In terms of space dimensions, the process appears to be faster in the buccal-lingual compared to the coronal-apical direction [6]. Several factors, such as number of residual alveolar walls, bone density, amount of periodontal bone loss, presence of infections and absence of adjacent teeth, seem to affect both time and pattern of resorption [7].

It is thus advisable to conserve original bone volumes and physiological ridge contours to guarantee an appropriate implant-supported prosthetic rehabilitation and to avoid complex surgical procedures [8], often performed to reach adequate esthetic and functional outcomes.

In this proposal, different advanced techniques (intra/extra-oral bone grafts, split-crest and sinus lift), not free from complications, were proposed [9,10] to restore proper volumes after post-extraction bone resorption, while other studies [11,12] suggested post-extraction ridge preservation. This objective can be obtained by filling the alveolus with biocompatible materials [13] to offset bone reduction following physiological bundle bone contraction. Considering the difficulty in raising autologous bone in the mouth and the consequent morbidity of this harvesting procedure [14], other biomaterials were proposed in the literature for this aim, e.g., homologous or xenologous bone graft [15,16] or resorbable synthetic materials [17,18], in association or not with a collagen membrane [19]. Experimented bone fillers showed promising results in terms of volumetric preservation of post-extraction sites if compared with the alveoli healed spontaneously [14,20,21].

Similarly to a physiological remodeling process, which has a main impact on the dimensional aspect, bone quality represents a further important factor for long-term stability when biomaterials are employed [22], especially in the case of implant placement. As predictability of success in bone characterized by a determined percentage of non-vital elements has not yet been determined, many clinicians usually prefer to possibly place the implant in bone structures characterized by a high regenerative potential, that is with a relevant number of cells [15,23].

Synthetic bone substitutes (resorbable, partially resorbable or not resorbable) usually ensure fast healing, being osteoconductive, easily available, and without toxicity, immune or infective risks [24,25,26]. Recent development of specific ones with highly osteoconductive properties, e.g., calcium sulphate [27], used alone or in association with xenografts, alloplasts, allografts or autografts, was addressed to improve the vital part of bone in post-extraction sites [28,29,30,31,32].

Deproteinized bovine bone is an anorganic osteoconductive material characterized by a chemical and structural architecture similar to human bone mineral [33] and able to maintain the space and stability during long periods of time because of its slow resorption rate and similar elastic modulus to the trabecular native bone.

Despite being found to enhance bioactivity (compared to synthetic hydroxylapatite), resorption of particles of this xenograft predominantly occur through osteoclastic activity, with residual graft particles present in grafted sites for up to 24 months [34]. Furthermore, particles placed adjacent to soft tissues and remote from host bone may simply become encapsulated with soft tissues and seem to be virtually resistant to resorption [35]. In this regard, the rationale of the association of deproteinized bovine bone with calcium sulphate relies on the rapid resorption of calcium sulphate (4 to 8 weeks), which encourages its use for socket grafting or implant site development not as a stand-alone but as a “binder” type of material, usually mixed with it to improve handling and to prevent particle migration [31]. In addition, calcium sulphate provides the release of ions, which creates acidity, thus providing antimicrobial properties [30].

In light of these considerations, the aim of this retrospective study was to evaluate the 4-month histological bone healing of post-extraction alveoli, filled with a graft composed of deproteinized bovine bone in association or not with calcium sulphate. The comparison between groups focused on the histomorphometry: this analysis, which is conceived as a non-invasive biopsy possible to perform as concomitant with implant placement, properly highlighted the earliest intense cellular bone variations of the first months after extraction.

## 2. Materials and Methods

### 2.1. Study Design: Retrospective Evaluation

The present study was designed and conducted in compliance with the principles of the Declaration of Helsinki on medical protocol and ethics and good clinical practice guidelines for research on human beings.

A retrospective study was conducted in 2023 on patients who had been previously referred for one or more mono-/pluri-radicular “hopeless” teeth, with one root needed for extraction. To be precise, the population was composed of patients recruited and treated in the context of a 4-month pilot study at the Dentistry and Maxillo-Facial Surgery Clinic of the University of Verona between October 2008 and June 2009.

A retrospective evaluation of the patients’ available medical written records was conducted in 2023, with collection and analysis of the data regarding clinical and histological outcomes of previously performed surgical procedures. In that context, the nature and aim of the surgical protocol of the pilot study, together with the anonymity in the scientific use of data, had been clearly explained in a written, informative consent form, which was signed by every patient at the time of surgery.

Ethical approval for the retrospective evaluation was requested and obtained from University of Verona Institutional Review Board in 2023 (protocol code “Prog.89CET BONE-REGENERATION”, 11 October 2023).

### 2.2. Inclusion Criteria: Retrospective Evaluation

The inclusion criteria were set to provide the following clinical and histological data, retrievable from patients’ medical records:

- Age more than 18 years old;
- Having had one or more mono-radicular o pluri-radicular “hopeless” teeth, with one root needed for extraction; alveoli without any apical infections or active periodontal disease; alveoli with more than one bony wall;
- Having had the alveoli grafted with a graft composed of deproteinized bovine bone in association with calcium sulphate, or deproteinized bovine bone alone;
- Having had the alveoli processed for the histomorphometry concomitant with implant placement 4 months after extraction.

On the other hand, patients with previous or current severe systemic diseases (ASA III and IV), pregnancy or breastfeeding, smokers, allergy/intolerance to bone materials used in the study or patients with clinical/histological missing data at the 4-month follow-up were excluded from the retrospective evaluation.

### 2.3. Description of Surgical Protocol and Grafting Materials: Retrospective Evaluation

From the analysis of the available medical records, it was found that the use of two different grafts in the sites treated in the pilot study was set as a randomized scheme: alveoli of patients had been randomly assigned (using a predefined computer-generated randomization scheme) to the test group, which received a graft composed of deproteinized bovine bone in association with calcium sulphate, or to the control group, which received only deproteinized bovine bone. For this scheme, Microsoft Excel Version 16.92 (24120731) was used to generate the random sequence, which was defined by even or odd numbers for the test group or for the control group, respectively. The sequence generation and proper allocation concealment were monitored by a dentist not involved in the participants’ enrollment. Opaque and sealed envelopes, each containing the secret code and bearing on the outside only a number, were opened after patients’ recruitment and signing of informed consent so that the investigator involved in the enrollment and treatment could not know in advance which treatment the next person was allocated.

**Figure 1:**
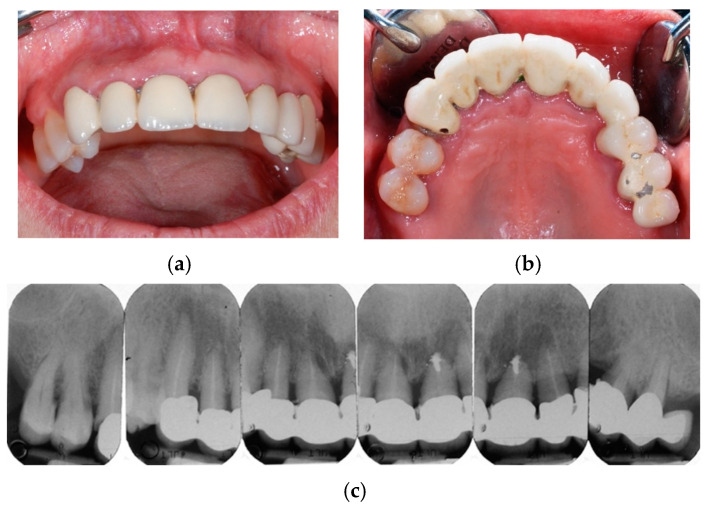
A 67-year-old woman presenting a fixed bridge in the upper maxilla from left second premolar to right canine: pre-operative frontal (**a**) and occlusal (**b**) clinical view; periapical X-rays of the upper maxillary elements (**c**): see multiple endodontic lesions. As visible from the radiographs, “hopeless” sites needed for extraction were 5, but the surgeon considered the extractions of all superior teeth for long-term prosthetic rehabilitation requirements.

**Figure 2:**
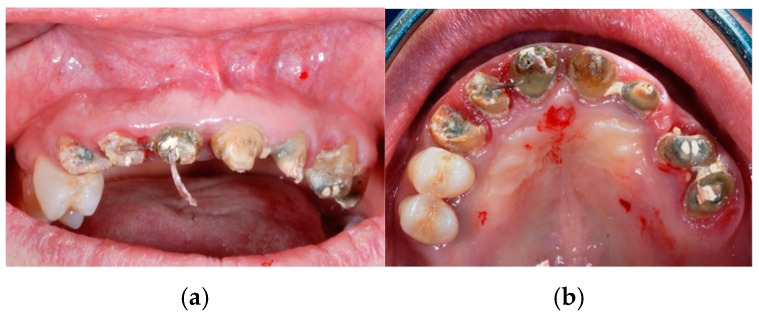
Removal of the fixed bridge before surgical extractions: see roots of the treated elements both from the frontal view (**a**) and the occlusal view (**b**).

**Figure 3:**
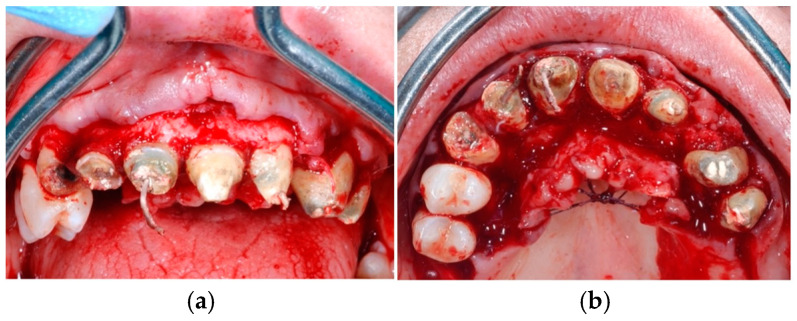
Surgical extractions with full-thickness mucoperiosteal flap elevation (**a**), aiming at preserving cortical alveolar walls (especially for the buccal side) with minimal trauma (**b**).

**Figure 4:**
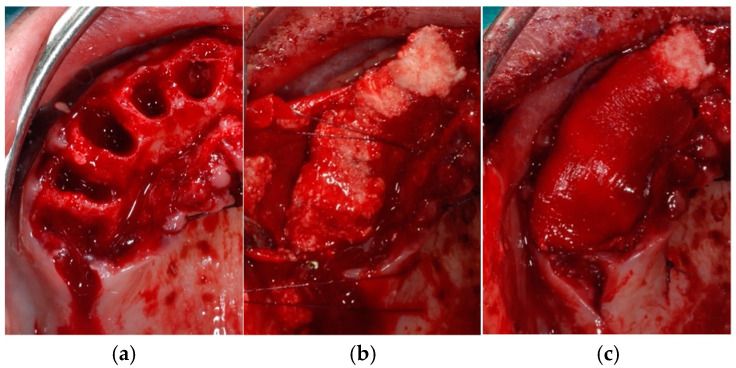
Sites of the test group (right side) (**a**) filled with deproteinized bovine bone in association with calcium sulphate (**b**), followed by the placement of a collagen membrane to cover the sites (**c**).

**Figure 5:**
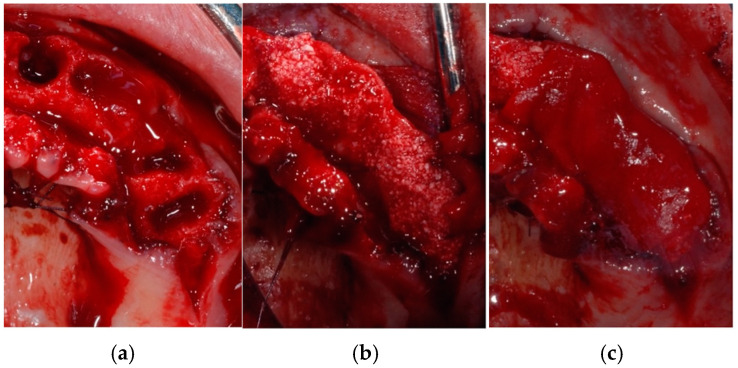
Sites of the control group (left side) (**a**) filled with deproteinized bovine bone alone (**b**), followed by the placement of a collagen membrane to cover the sites (**c**).

**Figure 6:**
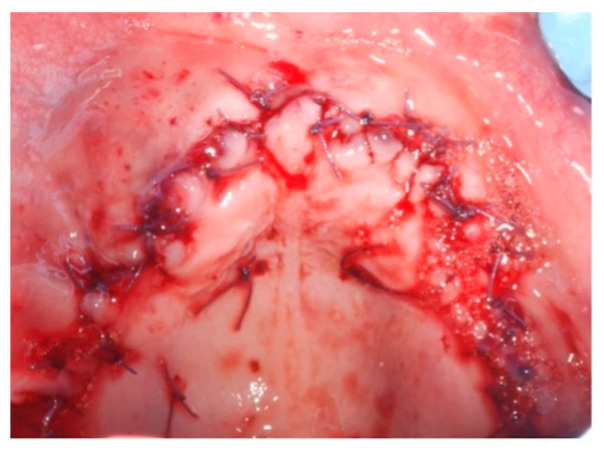
Final sutures (occlusal view).

**Figure 7:**
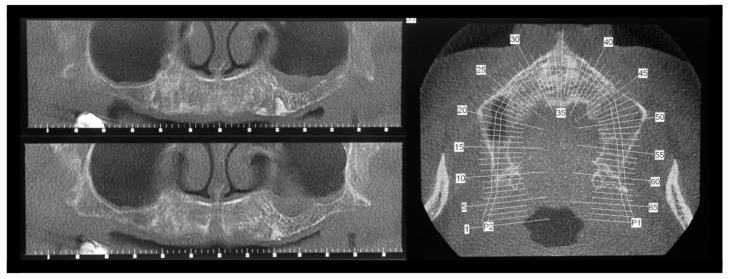
After 4 months, the patient was scheduled for implant placement, and a TC evaluation was performed: see more marked contour and radiopaque images of alveoli on the left side (control group) compared to the right side (test group).

**Figure 8:**
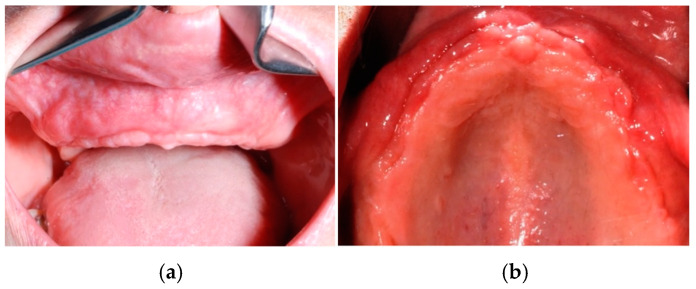
Clinical view (frontal in (**a**) and occlusal in (**b**)) of the upper maxillary arch before implant placement.

**Figure 9:**
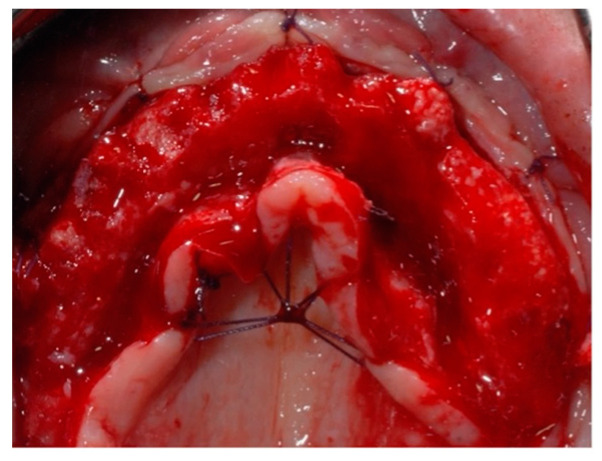
Full-thickness mucoperiosteal flap elevation.

**Figure 10:**
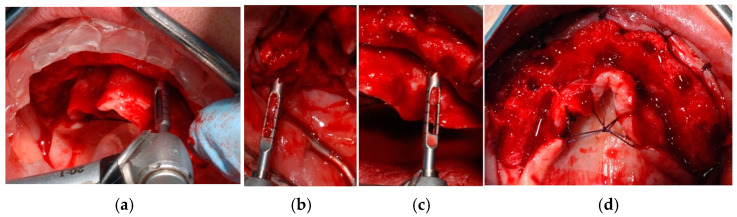
Using a dedicated core drill (**a**) with the aid of a surgical guide (**b**), biopsies of regenerated bone were performed in four sites for histomorphometric analysis of post-extractive sites (**c**,**d**).

**Figure 11:**
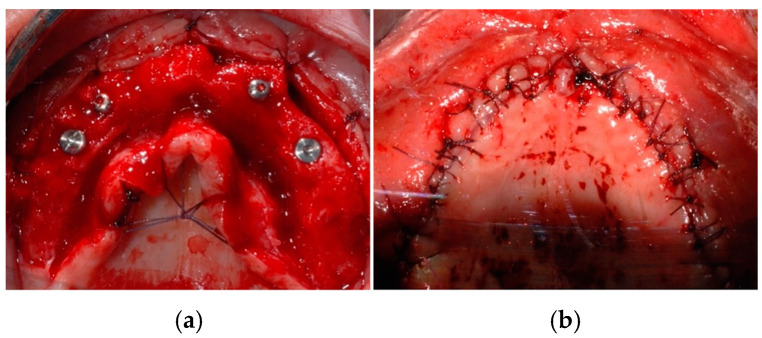
Implants placement (**a**) and final closure with sutures (**b**).

From the retrospective evaluation, it was also found that all patients had been treated by the same surgeon to minimize possible variability in procedures of extraction, grafting and implant placement (see Figure 1, Figure 2, Figure 3, Figure 4, Figure 5, Figure 6, Figure 7, Figure 8, Figure 9, Figure 10 and Figure 11). Preliminary evaluations consisted in standard clinical and radiographic examination (periapical X-rays and panoramic radiograph). After local anesthesia (infiltration with 1.8 mL mepivacaine 3% solution + adrenaline 1:100,000), a sulcular incision was made both on palatal/lingual and buccal side, followed by two vertical and buccal releasing incisions, to finally raise a full thickness mucoperiosteal flap. Teeth were extracted with an atraumatic procedure to avoid cortical alveolar bone fractures, using syndesmotome, extraction forceps and dental elevators [36]. Bone curettes were then used to remove granulation tissue from the alveolus.

Grafting materials were prepared as follows:

- Sites belonging to the test group were filled with deproteinized bovine bone (Bio-Oss^®^ Spongiosa Granules 0.25–1 mm, Geistlich Biomaterials, Wolhusen, Switzerland) mixed in a 50:50 proportion with an experimental preparation based on calcium sulphate (CalMatrix^®^ Calcium Sulfate Bone Graft Binder, Lifecore Biomedical, Chaska, MN, USA);
- Post-extractive alveoli of the control group were filled with deproteinized bovine bone, only mixed with a few drops of saline solution.

A collagen membrane (BioGide^®^; Geistlich Biomaterials, Wolhusen, Switzerland) was placed in both groups to cover the site, and the flap was sutured over it.

Post-surgical prescriptions consisted of antibiotics (amoxicillin + clavulanic acid, Augmentin^®^ cpr 1 g; GlaxoSmithKline, Verona, Italy) 1 g every 8 h for 5 days, in association with painkillers (OKI^®^, Dompé, L’Aquila, Italy) for the first 3 days, chlorhexidine 0.2% three rinses a day for 1 month and a soft diet for 2 weeks. Ice packs for the first 48 h were also suggested. All patients were visited 7 days after surgery, and sutures were removed after 14 days.

### 2.4. Description of Biopsies, Implant Placement and Histomorphometry: Retrospective Evaluation

From the analysis of the available medical records, it was found that after 4 months each patient had been scheduled to be treated for implant placement. After elevation of a full-thickness periosteal flap, a biopsy of regenerated bone was taken for histomorphometric analysis of post-extractive sites, using a 2.7 mm × 6.0 mm core drill (GA-33, Bontempi^®^; Tullingen, Germany). Implants were then placed, and sutures were performed. Patients followed the same abovementioned post-surgical instructions.

Histological analysis was performed in the Anatomical Pathology Laboratory at the University of Verona by a specialist in the field, again to minimize possible variability of examination.

**Figure 12:**
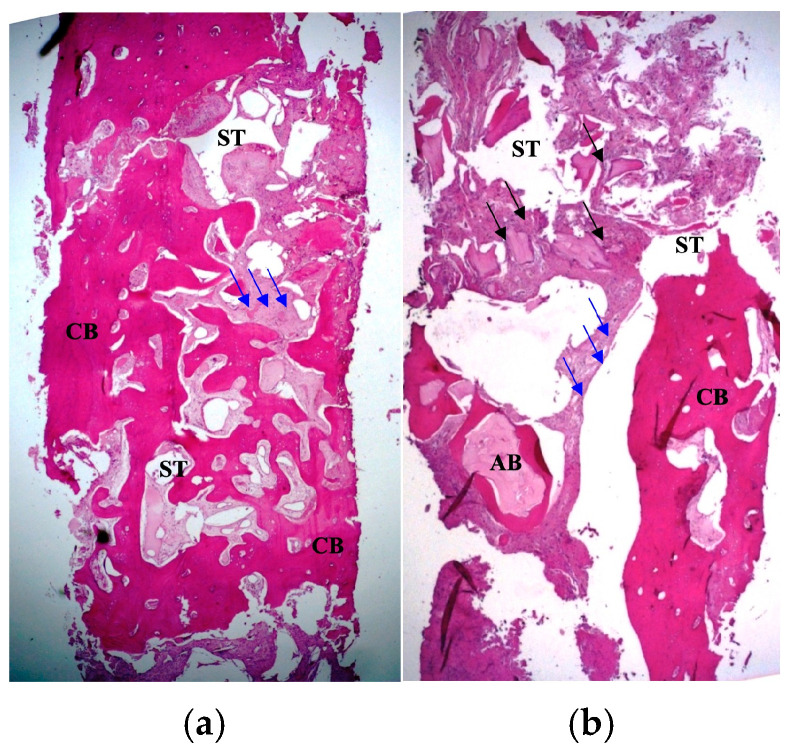
Results of histomorphometric analysis of 1.3 site: see prevalence of cellular bone (**a**). Results of histomorphometric analysis of 2.2 site: see prevalence of acellular bone (**b**). Cellular bone is identified as CB, acellular bone as AB, trabecular spaces as ST, residual grafted particles with black arrows and fibrous tissue with blue arrows.

Regarding histomorphometry, samples were first fixed in formalin 10% solution for 24 h and processed with a decalcificant liquid for 72 h, then included in paraffin to obtain central 5 µm width longitudinal sections using a microtome. One section was randomly selected for each sample by a second “blind” operator, processed and colored with hematoxylin–eosin and Schiff’s acid. Finally, histomorphometric analysis for qualitative evaluation was performed using microscopic light with 100–200× magnification, 10× lens and 10–20× reticulum. Image capture was performed using NIS Elements Imaging Software (Nikon Europe, Version 2.3) and Photometrics Coolsnap EZ camera (Photometrics, Tucson, AZ, USA). The ImageJ software program (Rasband, W.S., ImageJ, U.S. National Institutes of Health, Bethesda, MD, USA, Version 1.39p) was used for quantitative analysis, employing a measuring tool in conjunction with a magnification tool, entering the known calibrations for the microscope used to capture images: in this way, percentages of cellular bone (identified as CB), acellular bone (identified as AB), trabecular spaces (identified as ST), residual grafted particles (identified by black arrows) and fibrous tissue (identified by blue arrows) were respectively evaluated (see Figure 12).

Figure 1, Figure 2, Figure 3, Figure 4, Figure 5, Figure 6, Figure 7, Figure 8, Figure 9, Figure 10, Figure 11 and Figure 12 report a clinical case.

### 2.5. Statistical Analysis of Data Collected from the Retrospective Evaluation

For data collection and analysis, a database including all patients evaluated in the retrospective study was created with Microsoft Excel Version 16.92 (24120731). All data analysis was carried out using Stata v.13.0 for Macintosh (StataCorp, College Station, TX, USA). As the primary focus of the present evaluation was the 4-month histological comparison of post-extraction alveoli filled with two different grafts, site analysis was considered the most suitable in terms of the aim of the retrospective study design and considering the limitations of the available data (a small sample size) retrieved from the medical records. The normality assumptions for continuous data were assessed by using the Shapiro–Wilk test; mean and standard deviation were reported for normally distributed data, median and interquartile range (iqr) otherwise. For categorical data, absolute frequencies, percentages and 95% confidence intervals were reported. The association between categorical variables, also to compare groups for histomorphometry, was tested with a χ^2^ test; if any of the expected values was less than 5, a Fisher’s exact test was performed. The comparison of the means between two different groups was performed using unpaired Student’s “*t*” test. The level of significance was set at 0.05. The study presents compliance with the STROBE checklist guidelines [37].

## 3. Results

Relatively to the retrospective data collection, no patients presented missing data at the 4-month evaluation.

Of 20 patients declared as recruited and treated for the pilot study, they were all finally included for the retrospective evaluation. These patients, respectively, were 12 females and 8 males, aged between 35 and 67 years (average of 47.4 years of age).

As no complications had been reported during the healing period, and no evidence of adverse systemic or local reactions had been observed for each site, 24 bone samples were processed for the histomorphometry. Percentages of vital bone, acellular bone, trabecular spaces, residual grafted particles and fibrous tissue were measured for each examined biopsy section.

Twenty-four sites were overall analyzed, 12 for the test and 12 for the control group, respectively, to satisfy the inclusion criteria.

As shown in Table 1, the test group revealed significantly a higher quantity and smaller percentage of, respectively, cellular bone and acellular bone compared to the control group (*p* = 0.001).

Table 2 reports bone composition in both groups according to upper maxillae/mandibular sites: the test group revealed a significantly lower quantity of acellular bone, compared to the control group, in both arches (upper maxillae *p* = 0.001; mandible *p* = 0.04).

Table 3 reports bone composition in both groups according to posterior/anterior sites: the test group revealed a significantly higher quantity of cellular bone, compared to the control group, in both sectors (posterior *p* = 0.001; anterior *p* = 0.04).

## 4. Discussion

The extension and pattern of alveolar ridge resorption is considered an unavoidable consequence after tooth extraction, and it varies from individual to individual. A study by Carlsson et al. [38] evaluated a mean bone resorption of 23% of the original ridge volume six months after extraction. Shropp et al. [39], in a 1-year prospective evaluation, declared a more relevant width reduction (2/3 of the total bone loss) of 50% in the first 3 months after extraction, specifically in bucco-lingual and bucco-palatal aspects. Clinical and radiographic loss in width is declared in the literature [40,41] as a mean value of 3.87 mm, greater than a mean clinical mid-buccal height loss of 1.67 mm and a mean radiographic crestal height change of 1.53 mm. Moreover, a systematic review regarding soft tissue and bone dimensional changes of spontaneously healed human alveoli [1] reported a mean horizontal dimensional reduction of 3.79 mm and a mean vertical reduction of 1.24 mm on buccal aspect (0.84 mm on mesial and 0.80 mm on distal sites) 6 months after extraction, with values usually higher [42,43] compared to resorption on lingual/palatal walls.

It was observed [44] that the process of ridge resorption occurs in a differential way, more consistent and centripetal in the upper maxilla, while centrifugal in the mandible. Pietrokovski et al. [45] compared bone resorption in different edentulous areas, confirming the greatest bone resorption on the buccal surface compared to the lingual/palatal (especially of molars regions) and in upper maxillary arch compared to mandible. In addition, Serino et al. [7] demonstrated a better potential to regenerate lost or damaged walls in mandibular post-extraction sites.

The surgical protocol described in the present study for post-extraction ridge preservation is a technique proposed in the literature [11] to reduce physiological bone reabsorption in a post-extraction alveolus using graft materials. Deproteinized bovine bone was widely employed in the last decades in several investigations [15,28,46,47,48,49] because of its excellent biocompatibility, lack of adverse reactions, trabecular structure similar to human bone and good dimensional stability over time, with or without using membranes. Vance et al. [15] reported an average ridge width decrease of 0.5 mm 4 months after extraction using deproteinized bovine bone in association with a collagen resorbable membrane. Lindhe et al. [28], in a 6-month study with the use of the same graft, affirmed that it seems to efficiently limit post-extraction bone reabsorption, maintaining good dimensional stability, with a loss in horizontal thickness of only 1.6 mm. Jung et al. [47] used the same filling material and found a minimal resorption of the ridge volume, with a mean vertical defect fill of 91%. Other studies compared benefits of deproteinized bovine bone in terms of bone volume conservation compared to spontaneous healed sites, observing a buccal bone resorption less than 20% [50], while some authors [51] found that placement of the biomaterial in the fresh extraction socket retarded healing.

Once the importance of deproteinized bovine bone graft in avoiding alveoli resorption had been pointed out, several authors examined the quality of regenerated bone in post-extraction sites, reporting the ability of this material to develop new vital bone: percentages of 26% [15] and 20.7% [22] 4 months after extraction, and 26.9% [46], 26% [48], 30% [47] and 46.3% [52] 6 months after extraction were reported. Our analysis conducted on sites treated only with deproteinized bovine bone seems in agreement with the literature, with 31.25% of vital bone formation 4 months after extraction.

Nevertheless, the present study found a percentage of 62.5% in alveoli filled with deproteinized bovine bone in association with calcium sulphate: the authors thus suggest that deproteinized bovine bone alone does not seem to demonstrate high osteo-promotional qualities unless associated with other highly osteoconductive materials.

In addition, deproteinized bovine bone generally showed a steady and slow resorption pattern compared with other resorbable materials (e.g., demineralized freeze-dried bone allograft). Outcomes 4 months after healing reported a percentage of residual material of 16% [15] and 20.2% [22] despite the presence of new bone on the deproteinized bovine bone particles. Histomorphometric analysis performed in different investigations observed that the percentage of residual particles 6 months after extraction was even equal to 25.6% [46], 29.7% [53] and 30% [54], confirming a slow resorption time for this material [52] despite a progressive increase in bone formation. The control sites of the present study contained 8.6% of residual particles of material, a percentage which is quite inferior to the values listed above: if we consider it together with the acellular bone component, the overall value of mineralized amorphous component goes up to 41.5% of the total analyzed section, supporting the evidence for the poor osteo-promotional capability of deproteinized bovine bone alone.

Even though the long-term predictability of success of implants positioned in post-extraction ridges containing different percentages of residual inert particles of grafting material has not yet been determined, most authors declare that they prefer to place implants as much as possible in vital bone, with stable healing and regenerative potential [55]. In light of these considerations, different randomized trials tested the bone quality achieved with deproteinized bovine bone in association with other high osteoconductive grafting materials. Strocchi et al. [56] created bone defects, later filled with calcium sulphate granules or autologous bone, with half sites covered with a non-resorbable e-PTFE (polytetrafluoroethylene) membrane: an increase of micro-capillary density in defects treated with calcium sulphate was noticed, suggesting a positive effect on the angiogenesis process. Walsh et al. [57], after filling femoral bone defects with calcium sulphate particles, evidenced with immunofluorescence examination the presence of increased growing factors (bone morphogenetic protein-2, bone morphogenetic protein-7, transforming growth factor-beta and platelet-derived growth factor) in defects filled with calcium sulfate pellets alone and in combination with autograft, showing an essential role of these factors in the promotion of bone formation [47].

Even if these outcomes support the hypothesis that the used graft does not simply work as an inert filler [30,31], specific mechanisms promoting and optimizing osteogenesis still remain unknown, suggesting that the regenerative effect could be related to the proximity of graft particles to the adjacent bone, in combination with material’s capacity to guide new bone formation during its contemporary resorption. Toloue et al. [27] assessed an average of 32% and 16.7% new bone formation with 2.5% and 21% graft remaining, respectively, in alveoli filled with freeze-dried bone allograft or calcium sulfate. Vance et al. [15] used an allograft in an experimental putty carrier plus a calcium sulfate barrier or a bovine-derived xenograft plus a collagen membrane in human alveoli, assessing after 4 months greater vital bone fill (61% vs. 26%) in the first group, which may be attributable to earlier and greater vascular invasion of the carrier material, characterized by ease of handling, simple placement and enhanced graft particle containment. De Tullio et al. [58], performing a 5-month histomorphometric analysis on post-extraction sockets filled with calcium sulphate and sintered nano-hydroxyapatite, a combination of both or left to normal healing, found that the combined graft presented better behavior when compared to the individual application, with average percentages of vital bone of 13.56% for calcium sulphate, 17.84% for sintered nano-hydroxyapatite, 58.72% for the combined graft and 80.68% for the controls. To sum up, despite histological results of grafted sites sometimes overlapping with those obtained with spontaneous healing of alveoli, the prevalent advantages of all cited biomaterials in preserving ridge dimensions or, at least, in slowing down the post-extraction resorption process [26], can be evidenced overall.

As regards the clinical decision-making in socket preservation, procedures can be influenced not only in terms of choice of the most suitable material capable of maintaining the space during the regeneration process [59,60] but also in terms of the targeted bone quality and stability. According both to the literature and to the study findings, outcomes demonstrated that the addition of calcium sulphate to deproteinized bovine bone improves the vital bone formation in a relatively short time, not only in terms of early osteoconductive modifications but also for long-term stability of bone formation. Thus, the cost-effectiveness of using the combined graft, despite being more expensive compared to singular material, is crucial for clinical situations characterized by the need to obtain a stabilized graft effectively filling the interparticle spaces [61], together with bacteriostatic function and proper cell migration, preventing the invasion of soft tissues and encapsulation by fibrous tissue at the same time.

In addition, one of the components of CalMatrix^®^, sodium carboxy-methyl-cellulose, is a natural derivative of cellulose, a thickening and stabilizing additive, which allows the material to be easily positioned even in extensively reabsorbed alveoli. This is a particularly advantageous feature if compared to other formulations in granules or powder (e.g., Bio-Oss^®^) used alone and which are difficult to manage in non-contenitive and hemorrhagic defects.

### Study Limitations

Promising histological and clinical results of the present retrospective study encourage further prospective investigations with larger sample sizes and longer follow-ups. On the other hand, as it is a retrospective evaluation of a previous pilot protocol, its limitations should be identified.

First of all, retrospective studies inherently rely on the availability and accuracy of past records, which can influence the control over variables. In this proposal, data collection and analysis were performed in the best manner to avoid potential biases: data analysis focused on the primary focus of the evaluation, the histomorphometry, which was assessed as conducted through a detailed and standardized protocol by a specialist in the field.

The small sample size (only 24 sites in 20 patients) represents another limit mainly related to the retrospective study design: as no missing data were registered for the follow-up, the 4-month histological evaluation can be considered as complete on the available data retrieved from the medical records, with adequate handling of observations. Plus, even if more sites were evaluated in fewer patients, numbers of sites and of patients are superimposable, and only one patient presented multiple sites; thus, observations were not considered inadequately independent.

About the randomized scheme adopted at the time of surgery for the two grafts used, the randomization and allocation were accurately described in medical records, and the authors assumed them to be reliable for the outcomes observed, even in a retrospective context. Furthermore, even if the groups were homogeneous in site distribution, the comparison was conducted between only two groups: a multiple comparison between adjuvant graft materials could be advisable to properly deepen mechanisms of reabsorption.

Another critical issue is that the study does not mention any long-term follow-up after implant placement, which could be particularly important in studies involving surgical interventions and bone healing: the surgical protocol previously performed was designed as a pilot study which originally had the aim to be organized as a longitudinal study, but that finally was not continued. The rationale for taking into consideration these data after years is interest in the topic and type of biomaterials used to encourage further prospective studies: the retrospective evaluation represents part of a larger project with current and future updates.

Finally, the university hospital setting of the retrospective evaluation allowed for analysis of the available records found to have the precise inclusion/exclusion criteria: we underline that the findings are based on a specific patient population at a single institution, which may not be representative of broader patient populations.

## 5. Conclusions

Calcium sulphate, used in association with deproteinized bovine bone, was demonstrated to promote proper vital bone formation, with less acellular bone compared to deproteinized bovine bone used alone. As a significantly higher quantity of vital bone was revealed by the test group, this material seems to improve post-extraction ridge preservation in terms of histological aspects and regenerated bone quality. However, its properties as a resorbable grafting adjuvant need to be further investigated with a larger sample size and longer follow-ups.

## Figures and Tables

**Table 1:** Overall characteristics of 24 sites analyzed after sampling. All variables are expressed in n (%), or mean and standard deviation (n ± sd). Variables are also reported according to test/control groups.

	Overall [n (%), n ± sd]	Test Group [n (%), n ± sd]	Control Group [n (%), n ± sd]	*p* Value
**Sex**				
Male	8 (33.33)	4 (33.33)	4 (33.33)	0.66
Female	16 (66.67)	8 (66.67)	8 (66.67)	
**Arch**				
Upper maxilla	18 (75)	10 (83.33)	8 (66.67)	0.32
Mandible	6 (25)	2 (16.67)	4 (33.33)	
**Site**				
Posterior (premolar, molar)	18 (75)	10 (83.33)	8 (66.67)	0.32
Anterior (incisor, canine)	6 (25)	2 (16.67)	4 (33.33)	
**Bone composition**				
Cellular bone	46.87 ± 19.93	62.5 ± 15.3	31.25 ± 8.01	0.001 *
Acellular bone	18.95 ± 17.69	5 ± 3.01	32.91 ± 14.84	0.001 *
Trabecular spaces	27.66 ± 12.56	29.75 ± 15.7	25.58 ± 8.59	0.68
Residual grafted particles	6.08 ± 7.7	3.58 ± 14.59	8.58 ± 10.41	0.48
Fibrous tissue	0.83 ± 2.82	0.01 ± 0	1.66 ± 3.89	0.14

* = statistically significant difference between groups.

**Table 2:** Bone outcomes comparison between groups according to arch (upper maxilla/mandible). All variables are expressed in mean and standard deviation (n ± sd).

	Maxilla			Mandible			Maxilla vs. Mandible
	Test Group	Control Group	*p* Value	Test Group	Control Group	*p* Value	*p* Value
**Bone Composition**							
Cellular bone	60 ± 17.72	29.7 ± 7.61	0.001 *	67.5 ± 8.66	40 ± 0.01	0.06	0.06
Acellular bone	4.37 ± 3.2	34.5 ± 15.71	0.001 *	6.25 ± 2.5	25 ± 7.07	0.04 *	0.47
Trabecular spaces	33.62 ± 17.53	25 ± 9.35	0.24	22 ± 8.12	28.5 ± 2.12	0.14	0.56
Residual grafted particles	3.25 ± 1.41	9 ± 11.34	0.52	4.25 ± 1.5	6.5 ± 4.94	0.62	0.43
Fibrous tissue	0.01 ± 0	2 ± 4.21	0.19	0.01 ± 0	0.01 ± 0	0.1	0.4

* = statistically significant difference between groups.

**Table 3:** Bone outcomes comparison between groups according to sites (posterior/anterior). All variables are expressed in mean and standard deviation (n ± sd).

	Posterior Site			Anterior Site			Posterior vs. Anterior
	Test Group	Control Group	*p* Value	Test Group	Control Group	*p* Value	*p* Value
**Bone Composition**							
Cellular bone	62 ± 15.31	30.62 ± 9.42	0.001 *	65 ± 21.21	32.5 ± 5	0.04 *	0.54
Acellular bone	5.5 ± 2.83	29.37 ± 14.74	0.001 *	2.5 ± 3.53	40 ± 14.14	0.06	0.3
Trabecular spaces	29.8 ± 16.14	26.12 ± 7.79	0.92	29.5 ± 19.09	24.5 ± 11.26	0.64	0.78
Residual grafted particles	3.7 ± 1.51	11.37 ± 11.95	0.22	3 ± 1.41	3 ± 1.41	0.99	0.22
Fibrous tissue	0.01 ± 0	2.5 ± 4.62	0.1	0.01 ± 0	0.01 ± 0	0.1	0.4

* = statistically significant difference between groups.

## Data Availability

The data presented in this study are available upon request from the corresponding author.
